# Quantifying the dynamics of rocky intertidal sessile communities along the Pacific coast of Japan: implications for ecological resilience

**DOI:** 10.1038/s41598-021-95348-1

**Published:** 2021-08-09

**Authors:** Ken Ishida, Michikusa Tachibana, Masakazu Hori, Takehiro Okuda, Tomoko Yamamoto, Masahiro Nakaoka, Takashi Noda

**Affiliations:** 1 Graduate School of Environmental Science, Hokkaido University, N10W5, Kita-ku, Sapporo, Hokkaido 060-0810 Japan; 2 Fisheries Resources Institute, Japan Fisheries Research and Education Agency, 2-12-4, Fukura, Kanazawa-ku, Yokohama, 236-8648 Japan; 3 Faculty of Fisheries, Kagoshima University, 4-50-20, Simoarata, Kagoshima, 890-0056 Japan; 4 Akkeshi Marine Station, Field Science Center for Northern Biosphere, Hokkaido University, Aikappu, Akkeshi, Hokkaido 088-1113 Japan; 5 Faculty of Environmental Earth Science, Hokkaido University, N10W5, Kita-ku, Sapporo, Hokkaido 060-0810 Japan

**Keywords:** Community ecology, Community ecology, Marine biology

## Abstract

Long-term patterns in trajectories of natural communities provide insights into ecological resilience, but their assessment requires long-term census data. We analyzed 16-year census data for intertidal communities from 30 rocky shores along Japan’s Pacific coast to assign community change to four possible trajectories (stable, reversible, abrupt, or linear) representing different aspects of ecological resilience, and to estimate multiple metrics of temporal invariability (species richness, species composition, and community abundance). We examined (1) how the prevalence of the four trajectories differs among regions, (2) how the features (model coefficients) of each trajectory vary among regions, and (3) how the temporal invariabilities differ among trajectories and regions. We found that the stable trajectory was the most common. Its features differed among regions, with a faster recovery to steady-state equilibrium in low-latitude regions. Furthermore, trajectories and temporal invariabilities both varied among regions, seemingly in association with the strength of ocean current fluctuations. Thus, the relationship between community temporal invariability and trajectory may be weak or absent, at least at the regional scale.

## Introduction

**Figure 1:**
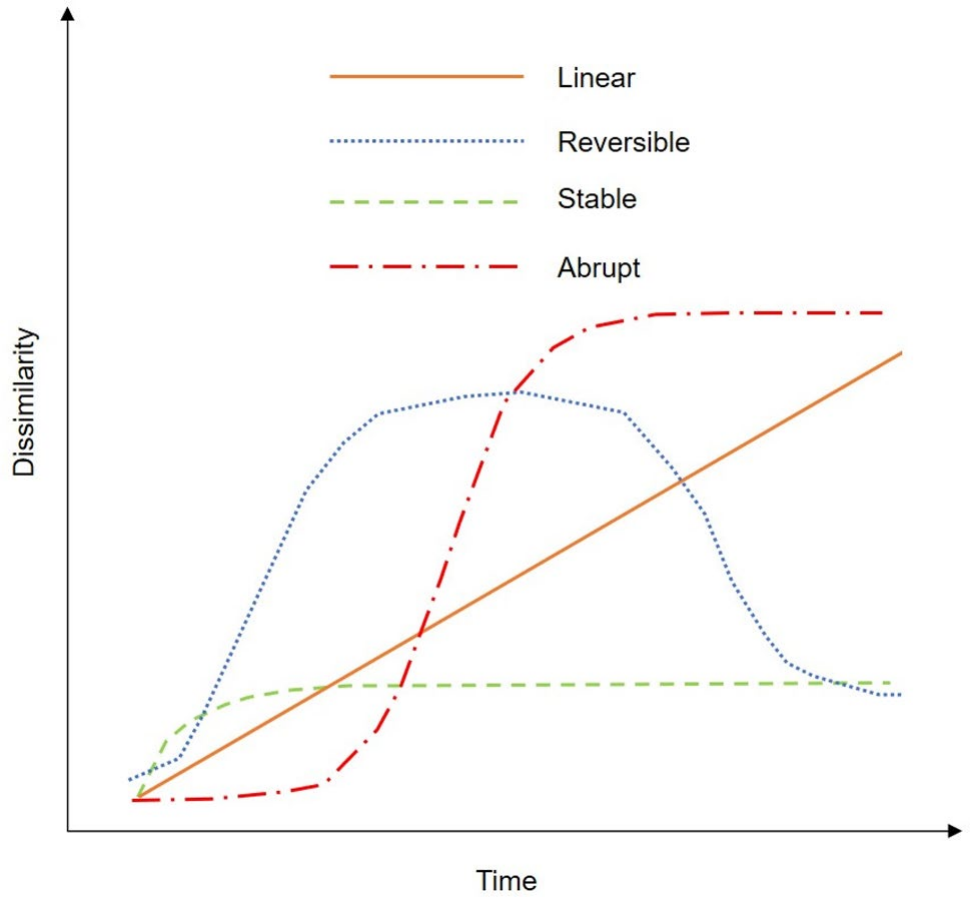
Conceptual diagram of four trajectories of community change (Bagchi et al. 2017)^5^. The *y*-axis represents the dissimilarity index of species composition between an initial time and each subsequent time. Each trajectory represents a different concept of community ecological resilience.

The composition of biological communities generally varies temporally because of environmental changes and ephemeral disturbances, the magnitude and frequency of which vary with location. It is therefore crucial for the proper management and conservation of ecosystems to quantify patterns of community trajectories and to assess their spatial variability. Understanding patterns of community trajectories also provides insights into ecological resilience—that is, the movement of a community within and between stable domains^1–4^—which is useful for community conservation and for predicting the potential of a community to recover from various disturbances. Bagchi et al. (2017)^5^ developed an empirical method that allows researchers to categorize the dynamics of a focal community into one of four trajectories: stable (i.e., species composition does not undergo any appreciable change over time), abrupt (a relatively sudden change in community composition), reversible (the community undergoes major changes in composition and later returns to the original state), and linear (slow, incremental change in community composition over time) (Fig. 1, Table 1).

**Table 1:** Four community trajectories used in this study and their corresponding concepts of ecological resilience as quantitatively identified by Bagchi et al. (2017)^5^.

Community trajectory	Description	Corresponding concepts of ecological resilience
Linear	Slow, incremental change with time	Nonequilibrium, phase shift
Reversible	Major changes, with return to original state	Engineering resilience
Stable	No appreciable change over time	Resistance, or asymptotic state
Abrupt	Relatively sudden change	State transition, threshold, regime shift, tipping point

The stable trajectory is defined by using an asymptotic model from the onset of movement away from a community defined as the baseline community as

1 $$\left( {{\text{SDI}}} \right)_{t} = \phi_{{1}} [{1 } - (\phi_{{2}} t)],$$

where (SDI)_*t*_ is the Sørensen dissimilarity index relative to the baseline community at time *t*, ϕ_1_ is the asymptote at steady-state equilibrium, and ϕ_2_ is the logarithm of the rate constant.

The abrupt trajectory is defined by a logistic model as

2 $$\left( {{\text{SDI}}} \right)_{t} = \frac{{\updelta }}{{1 + exp\left( {\frac{{{\uptheta } - t}}{\phi }} \right)}},$$

where δ is asymptotic height, θ is the time at which divergence (i.e., deviation from the baseline community) reaches half of δ, ϕ is the time between reaching one-half and three-quarters of the maximum divergence from the baseline community, and *t* is the time since the beginning of the record.

The reversible trajectory is defined by a double sigmoid mathematical function that estimates the distance, timing of onset and return phases (θ_o_ and θ_r_, respectively), and duration of onset and return (ϕ_o_ and ϕ_r_, respectively) as

3 $$\left( {{\text{SDI}}} \right)_{t} = \frac{{{\updelta }_{o} }}{{1 + exp\left( {\frac{{{\uptheta }_{o} - t}}{{\phi_{o} }}} \right)}} - \frac{{{\updelta }_{r} }}{{1 + exp\left( {\frac{{{\uptheta }_{r} - t}}{{\phi_{r} }}} \right)}}.$$

Here δ_o_ and δ_r_ are the asymptotic heights for onset and return phases of community change, θ_o_ and θ_r_ are the times of onset and return phases at which change reaches one-half of its asymptotic height, and ϕ_o_ and ϕ_r_ are the times elapsed between reaching one-half and three-quarters of the change distance for onset and return phases, respectively. *t* is the time interval from the beginning of the record.

Finally, the linear trajectory is defined with a linear model as

4 $$\left( {{\text{SDI}}} \right)_{t} = \alpha t + \varepsilon ,$$

where α is the diffusion constant, *t* denotes the time since the beginning of the record, and ε is an error term representing the intercept. These four trajectories provide a quantitative basis to compare and interpret ecological resilience, because each trajectory is related to distinct concepts of ecological resilience. However, trajectory analysis requires a long-term census of the community^5^.

In contrast, temporal invariability of a community can be relatively easily measured. For example, it doesn’t require long-term census, many temporal replicates and fitting to mathematical models, and it has been quantified for both aggregative properties (e.g., community biomass) and non-aggregative properties (e.g., community composition) for various communities, depending on the research topic and question^6–8^. In this study, we defined temporal invariability as the coefficient of variation (CV; aggregative properties) or mean (non-aggregative properties) of a variable over time^9^. Both ecological resilience and temporal invariability are components of community stability, which is a multidimensional concept^7–13^. The relationship between components of community stability may depend on the properties by which the components of stability are measured^7,8^. Therefore, elucidating how community trajectories covary with temporal invariability, which has been quantified for various community properties, across space provides a good opportunity to deepen our understanding of the dimensionality of community stability. Also, it provides insights of efficient strategies for evaluating the community trajectories. For example, it is reasonable to predict that the temporal invariability for communities with a stable trajectory is higher than for those with other trajectories.

There are several fundamental questions related to the spatial variation of trajectories and temporal invariability of a biological community. First, do the prevalences of the four trajectories vary spatially? Second, do the features (model coefficients) of these trajectories vary spatially? The answers to these two questions will improve our understanding of the consistency and variability in both quantitative and qualitative features of the trajectories of natural biological communities. Third, how do temporal invariabilities change among different trajectories or regions? Addressing this question should provide knowledge useful for predicting the trajectories of natural communities, understanding multidimensionality of stability and the interdependencies between components.

Rocky intertidal sessile communities are some of the best study systems for examining spatial variation of trajectories and temporal invariability. First, intertidal rocky shores are common and accessible habitats and have a suite of usually well-described and easily identifiable species^14^. Second, temporal changes in a rocky intertidal sessile community can be easily and non-destructively quantified as presence-or-absence and percent-coverage data at the same site because of the organisms’ sessile growth habit. In addition, the community dynamics occurs at a tractable time scale because the component species of rocky intertidal communities have relatively short life spans. Previous studies demonstrated that the pace of community dynamics^15,16^ and the strength of its determinant factors, such as larval flux, growth rate, and the strength of interspecific interactions, varied spatially depending on the coastal oceanography, such as intensity and frequency of upwelling^17–26^.

Here, we examined how the community dynamics of rocky intertidal sessile assemblages on the steep slopes of rocks vary spatially along the Northwestern Pacific coast of Japan (between 31°N and 43°N) by explicitly incorporating hierarchical spatial scale into the monitoring design. We specifically investigated (1) how the relative prevalence of four trajectories differs among regions, (2) how the features of each trajectory vary among regions, and (3) how the temporal invariability of species richness, species composition, and community abundance differs among trajectories or regions, or both. Specifically, we hypothesized that regional differences in these trajectories and in the community invariability of rocky intertidal sessile community dynamics along the Pacific coast of Japan are governed by spatiotemporal variability of the ocean current systems. The study area is in the Kuroshio–Oyashio transition area, where the warm Kuroshio Current meets the cold Oyashio Current, and there are marked differences in abiotic environmental factors between these currents^27–30^. The differences in environmental factors that reflect the current systems would likely cause differences in patterns of community dynamics.

Both the Kuroshio and Oyashio Currents show spatial and temporal fluctuations^27–33^. Previous studies have suggested that spatiotemporal variability of these ocean current systems has likely caused changes in dominant species^34,35^, recruitment strength^34,36,37^, mortality^35,38,39^, and abundance of coastal organisms^34–38^. Therefore, we speculate that increasing spatiotemporal variability of ocean current systems increases the relative frequencies of the linear, reversible, and abrupt trajectories and some model coefficients of each trajectory (α of the linear, δ_o_ and δ_r_ of the reversible, ϕ_1_ of the stable, and δ of the abrupt trajectory) and decreases the relative frequency of the stable trajectory and three measures of community temporal invariability: species richness, species composition, and community abundance.

## Results

**Figure 2:**
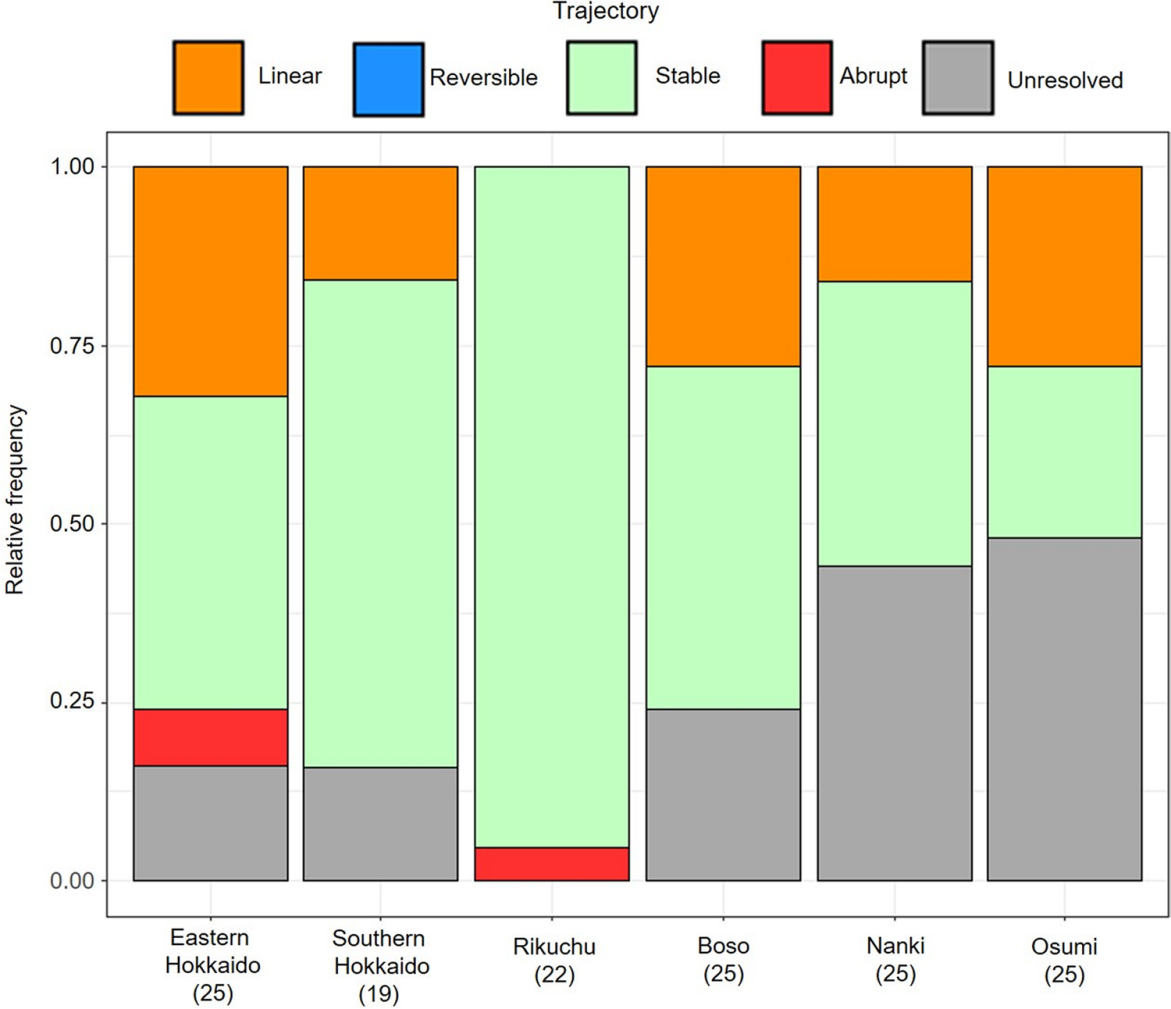
Relative frequencies of trajectories representing the patterns of ecological resilience of rocky intertidal sessile communities along the Pacific coast of Japan, based on the Sørensen dissimilarity index. Also shown are the relative frequencies of instances where trajectories could not be reliably classified (i.e., “unresolved”). The numbers of plots in each region are in parentheses.

The stable trajectory was the most common of the four trajectories, followed by the linear trajectory (except at Rikuchu and Osumi). The abrupt trajectory was detected only in eastern Hokkaido and at Rikuchu, and the reversible trajectory was not detected (Fig. 2). The proportions of trajectories differed significantly among regions (Fisher’s exact test, *P* < 0.001).

All coefficients (α, a coefficient of the linear trajectory, and ϕ_1_ and ϕ_2_, coefficients of the stable trajectory) estimated from the best-fit models varied significantly among regions (Supplementary Table S1 online). At the sites with linear dynamics, the Osumi community was more invariant than the Nanki community (parameter α; Table 2). At the sites with stable dynamics, community stability around the steady-state equilibrium in southern Hokkaido was higher than that of other regions (parameter ϕ_1_) and the recovery rates to steady-state equilibrium in the Nanki and Osumi communities were faster than those of other regions (parameter ϕ_2_; Table 2). Multiple regression analyses suggest that parameters α and ϕ_1_ were higher with stronger current fluctuations, and parameter ϕ_2_ was lower in areas under the influence of the Kuroshio Current (Table 3).

**Table 2:** Summary of model coefficients (mean ± SD) for each trajectory found for communities of rocky intertidal sessile organisms in six regions along the Pacific coast of Japan.

Region	Linear	Stable	Abrupt
Eastern Hokkaido	α = 0.014^ab^ ± 0.004	φ_1_ = − 0.34^bc^ ± 0.09	δ = 0.26 ± 0.09
		φ_2_ = − 0.69^a^ ± 0.45	θ = 2.32 ± 0.62
			φ = 1.34 ± 0.09
Southern Hokkaido	α = 0.012^ab^ ± 0.004	φ_1_ = − 0.32^c^ ± 0.04	NA
		φ_2_ = − 0.92^ab^ ± 0.36	
Rikuchu	NA	φ_1_ = − 0.48^a^ ± 0.06	δ = 0.41 ± 0
		φ_2_ = − 0.66^a^ ± 0.47	θ = 1.62 ± 0
			φ = 0.66 ± 0
Boso	α = 0.012^ab^ ± 0.006	φ_1_ = − 0.39^bc^ ± 0.05	NA
		φ_2_ = − 0.83^ab^ ± 0.34	
Nanki	α = 0.017^a^ ± 0.003	φ_1_ = − 0.41^ab^ ± 0.06	NA
		φ_2_ = − 1.29^b^ ± 0.29	
Osumi	α = 0.008^b^ ± 0.002	φ_1_ = − 0.39^abc^ ± 0.09	NA
		φ_2_ = − 1.41^b^ ± 0.54	

Different superscript letters signify statistically significant differences as shown by post hoc Bonferroni pairwise comparisons (*P* < 0.05). NA (not available) indicates that a trajectory was not applicable in a particular region.

**Table 3:** Results of multiple regression analysis for the effects of current systems and strength of current fluctuations on model coefficients of each trajectory type (linear [α]; stable [ϕ_1_, ϕ_2_]) and on three measures of community temporal invariability (species richness, species composition, and community abundance).

Response variable	Explanatory variable	Estimate	Std. error	t-value	*P*
Linear α	Intercept	0.0057	0.0026	2.19	0.0377
	Current systems	0.0037	0.0019	1.94	0.0639
Transformation: none	Current fluctuation	0.0033	0.0013	2.59	0.0157
Stable φ_1_	Intercept	0.2785	0.0288	9.66	< 0.001
	Current systems	− 0.0024	0.0188	− 0.13	0.9000
Transformation: none	Current fluctuation	0.0553	0.0116	4.78	< 0.001
Stable φ_2_	Intercept	− 1.1808	0.1691	− 6.99	< 0.001
	Current systems	0.3777	0.1100	3.44	< 0.001
Transformation: none	Current fluctuation	0.0281	0.0679	0.41	0.6804
Species richness	Intercept	0.8254	0.0349	23.67	< 0.001
	Current systems	− 0.0415	0.0241	− 1.72	0.0886
Transformation: log_10_	Current fluctuation	− 0.0499	0.0148	− 3.37	0.0011
Species composition	Intercept	− 0.1749	0.0110	− 15.85	< 0.001
	Current systems	0.0223	0.0076	2.92	0.0043
Transformation: log_10_	Current fluctuation	− 0.0360	0.0047	− 7.69	< 0.001
Community abundance	Intercept	0.8981	0.0808	11.12	< 0.001
	Current systems	− 0.2539	0.0559	− 4.54	< 0.001
Transformation: log_10_	Current fluctuation	0.0693	0.0343	2.02	0.0461

Columns report the estimated coefficients for explanatory variables, their standard errors, t-values and *P*-values.

There was a significant interaction between region and trajectory for the temporal invariability of species richness. However, neither the temporal invariability of species composition nor community abundance varied significantly among trajectory types (Table 4).

**Table 4:** Results of two-way ANOVA for the effects of region and trajectory (linear or stable)^a^ on three measures of community temporal invariability (species richness (a), species composition (b), and community abundance (c)).

Source of variation	df	MS	*F*	*P*
**(a) Species richness**				
Region	5	0.05431	4.209	0.00174
Trajectory^a^	1	0.00417	0.323	0.57113
Region × Trajectory	4	0.05376	4.166	0.00383
Residuals	91	0.01291		
Transformation: log_10_				
Levene's test: *F* = 1.7328, *P* > 0.05				
**(b) Species composition**				
Region	5	0.020171	13.978	< 0.001
Trajectory^a^	1	0.000529	0.366	< 0.5464
Region × Trajectory	4	0.002939	2.037	< 0.0958
Residuals	91	0.001443		
Transformation: log_10_				
Levene's test: *F* = 1.5378, *P* > 0.05				
**(c) Community abundance**				
Region	5	1.1666	29.392	< 0.001
Trajectory^a^	1	0.0213	0.537	< 0.465
Region × Trajectory	4	0.0684	1.725	< 0.151
Residuals	91	0.0397		
Transformation: log_10_				
Levene's test: *F* = 0.7907, *P* > 0.05				

^a^Please see main text for a description of community trajectories.

**Figure 3:**
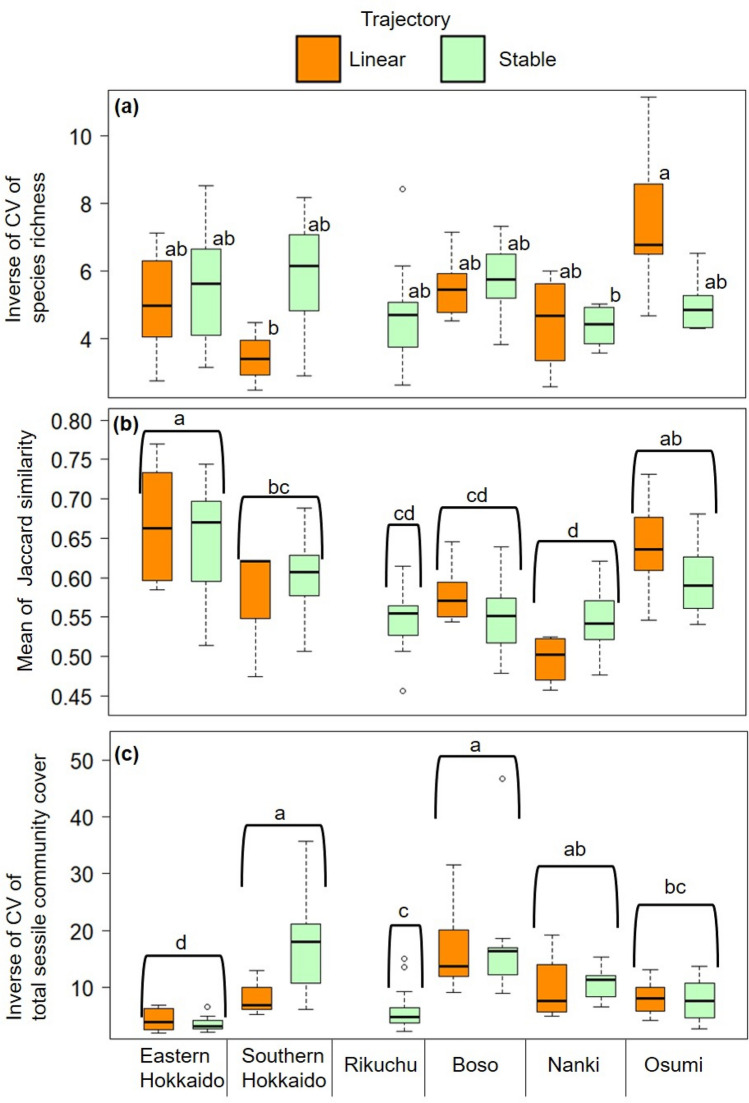
Box plots of the temporal invariability of (**a**) species richness, (**b**) species composition, and (**c**) community abundance. Box plots show the median, minimum, maximum, and first and third quartiles. Different letters indicate statistical differences in post hoc Bonferroni pairwise comparisons at *P* < 0.05.

The temporal invariability of species richness, species composition, and community abundance varied among regions (Table 4). The species richness of communities in Osumi classified as having linear trajectories was more invariable than that of communities in southern Hokkaido with linear trajectories and those in Nanki with stable trajectories (Fig. 3a). The species composition of communities in eastern Hokkaido and Osumi were significantly more stable than those in other regions (Fig. 3b). Community abundance at sites in southern Hokkaido and Boso was more stable than that in other regions (Fig. 3c).

Current fluctuation reduced the temporal invariability of both species richness and species composition while increasing the temporal invariability of community abundance (Table 3). Kuroshio Current regions exhibited lower temporal invariability of species composition but higher temporal invariability of community abundance than Oyashio Current regions (Table 3). Significant correlations were detected among the three measures of temporal invariability. The mean of the Jaccard similarity between consecutive years was positively correlated with the inverse of the CV of species richness, whereas it was negatively correlated with the inverse of the CV of community abundance (Supplementary Fig. S1 online).

## Discussion

Our study area is located in the Kuroshio–Oyashio transition area, in which the Oyashio is characterized by low temperature, low salinity, and high primary productivity and the Kuroshio is characterized warmer temperatures, higher salinities, and generally low primary productivity^27–30^. Previous studies in the area have shown that species richness in intertidal sessile assemblages within each region decreases with increasing latitude^40^, and a cluster analysis of similarities showed that the rocky intertidal assemblages could be first classified overall into two groups (comprising the three northern regions and the three southern regions), and then further separated into distinct groups for each region, except for the two southern regions (Nanki and Osumi)^41^. Despite these previously reported regional differences, we found a consistent pattern in the proportional distribution of trajectories across regions. The stable trajectory was the most common, followed by linear, whereas the other trajectories were rarely detected. A similar pattern has been observed in perennial grass communities, where stable and linear trajectories were the first and second most common, respectively^5^. In plant and animal communities across the globe, the majority exhibit regulated fluctuations of both species richness and total abundance^42^. In addition, both within and across meta-analyses that include terrestrial and aquatic systems, threshold transgressions are rarely detectable^43^. These studies suggest that biological communities are generally regulated. Furthermore, the trajectories in common between rocky intertidal sessile assemblages and perennial grass communities at the regional scale suggest that some sessile communities in marine benthic and terrestrial habitats show stable or linear dynamics. Testing this hypothesis will require more research on the spatial variation of trajectories in various habitats and taxa.

We did find a significant regional difference in the relative frequency of trajectories (Fisher’s exact test; *P* < 0.001). Interestingly, the frequency of the stable trajectory per plot was the highest at Rikuchu, despite this site experiencing subsidence and a large tsunami during the 2011 Great East Japan Earthquake. This result suggests that the impact of the March 2011 earthquake on rocky intertidal sessile community dynamics was relatively small^44^. Although the earthquake obviously affected the population size of some sessile species^45,46^, it did not significantly affect the dynamics of species composition in the local communities.

The linear trajectory is defined by using a linear model in which α is the diffusion constant; the stable trajectory is defined by using an asymptotic model in which ϕ_1_ is the asymptote at steady-state equilibrium and ϕ_2_ is the logarithm of the rate constant^5^. α and ϕ_1_ increased with greater current fluctuation. These results appear to support the idea that variation in environmental conditions influences species composition^47^. ϕ_2_ was lower for communities influenced by the Kuroshio Current (Nanki and Osumi in particular) than for those influenced by the Oyashio Current and in other regions. This suggests a faster recovery to steady-state equilibrium in regions at lower latitudes, presumably because marine organisms living in warmer environments are faster growing and shorter lived^48,49^.

We found that the relationship between community trajectories and temporal invariability was absent or weak. The trajectory analysis method that we used^5^ provides an accurate and robust method for distinguishing community dynamics that provides insights into characteristics of ecological resilience and their interpretation; however, it requires long-term census data for the community, many temporal replicates and fitting to mathematical models. In contrast, community temporal invariability is easier to estimate. Therefore, if a community trajectory could be estimated from the community temporal invariability, it would not only advance our understanding of the multidimensional nature of community stability^9–11,13^ but would also greatly improve our ability to establish plans for the sustainable use and conservation of ecosystems by predicting natural community dynamics. Here, we examined whether there is a relationship between these trajectories and temporal invariability. Our results did not show any clear relationship, at least for rocky intertidal sessile communities, indicating that direct estimation is necessary to identify the trajectories of a community. To clarify this issue, further research is necessary on the relationship between community temporal invariability and ecological resilience for rocky intertidal sessile communities and for other community types.

Temporal invariability of species richness, species composition, and community abundance are components of stability that characterize community dynamics. Therefore, understanding their spatial variation is crucial for the evaluation and management of ecosystems. Here, we detected significant differences between regions and between ocean current systems in the temporal invariability of species composition and community abundance of rocky intertidal sessile communities. The temporal invariability of biomass of an algal assemblage was shown to decrease with increasing latitude along the European coast^50^. This pattern could result from a decrease in the mean value or an increase in the standard deviation of the aggregate property, or both^50^. Our results show that the temporal invariability of community abundance was less stable near the Oyashio Current (that is, at higher latitudes) than near the Kuroshio. However, it did not show an explicit trend along a latitudinal gradient. As expected, the effect of spatiotemporal fluctuations in the flow patterns of ocean currents emerged in the temporal invariability of species richness, species composition and community abundance of rocky intertidal sessile communities.

## Conclusions

The present study examined how community dynamics of intertidal sessile assemblages on steep rocky slopes vary spatially along the Northwestern Pacific coast of Japan between 31°N and 43°N. The majority of communities exhibited stable dynamics in species composition (i.e., species composition did not undergo any appreciable change) during the 16 years studied. We found no clear interdependence between the community trajectories and quantified temporal invariabilities for species richness, species composition, or community abundance. Therefore, direct estimation is necessary to identify the trajectories of a community. Regional differences were detected in both trajectories and temporal invariabilities, associated with spatiotemporal variability of the ocean current systems in this area. Future research is needed to elucidate the linkage between spatiotemporal variability of the ocean current systems in the Kuroshio–Oyashio transition area and the dynamics of rocky intertidal sessile communities.

## Materials and methods

### Census design

**Figure 4:**
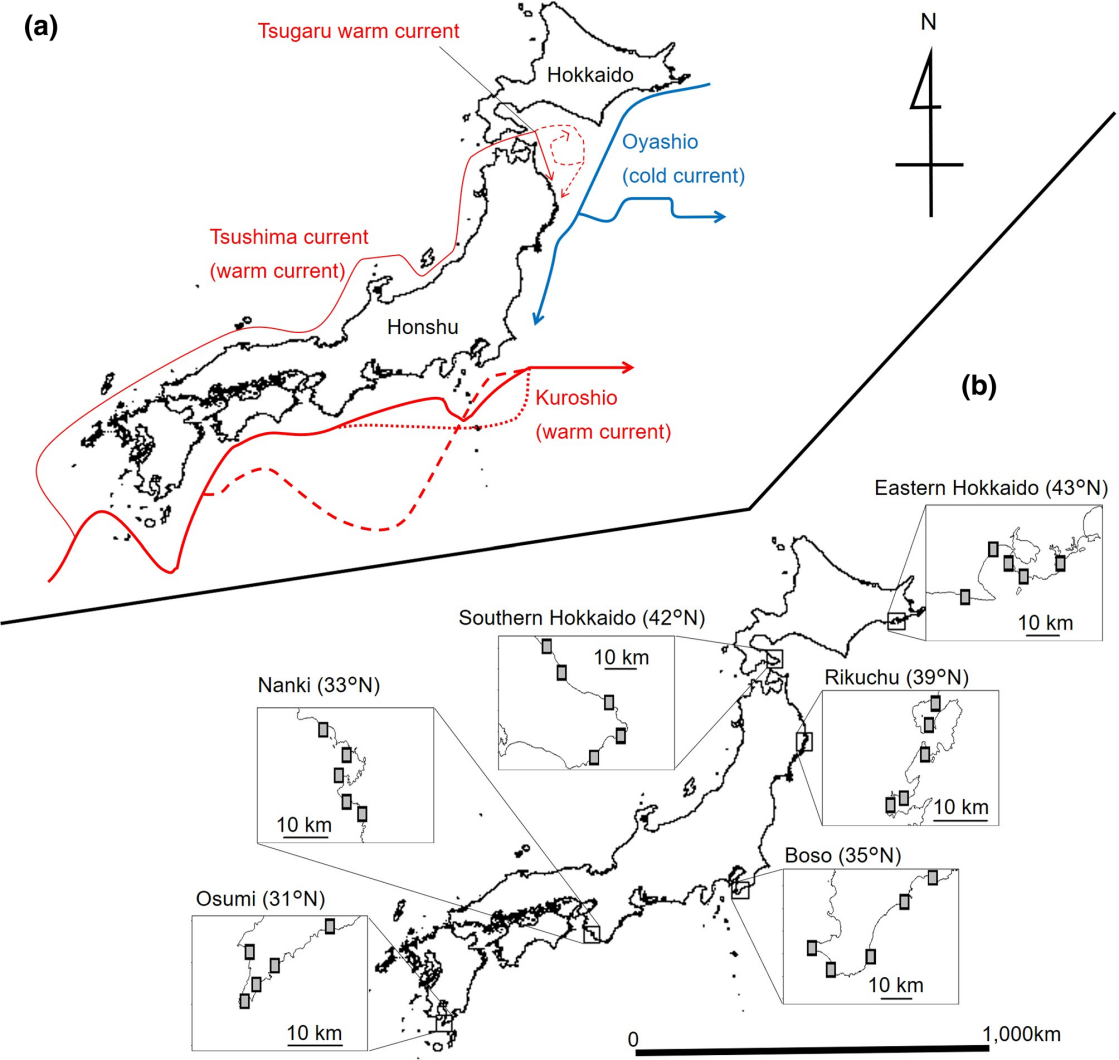
Maps showing (**a**) major ocean current systems around Japan, and (**b**) the six study regions along the Northwestern Pacific coast of Japan between 31°N and 43°N. Five rocky shores (shaded squares) were chosen for the census of intertidal sessile organisms in each region. The red solid and broken lines in (**a**) show alternative current paths. This figure was generated with R version 3.5.2 and the GADM database (www.gadm.org), version 2.5.

We used hierarchical nested sampling^51^ for the layout of each site. Five rocky shores were chosen for the census of intertidal sessile organisms in each of six regions (eastern Hokkaido, southern Hokkaido, Rikuchu, Boso, Nanki, and Osumi; Fig. 4) along the Pacific coast of Japan between latitudes 31°N and 43°N, with intervals between neighboring regions of 263–513 km (mean ± SD: 404.9 ± 107.3 km)^52^. Coastal marine biota in the area are affected by two major current systems: the warm Kuroshio along the southwestern coast and the cold Oyashio from the eastern coast of Hokkaido to the northeastern coast of Honshu (Fig. 4a)^27,29,30^. The three northern regions are under the influence of the Oyashio Current, with conditional influence from the Tsugaru Warm Current, a branch of the Kuroshio, to southern Hokkaido and Rikuchu^29,53–56^. The Oyashio and the Tsugaru Warm Current show interannual variations; the southern limit of Oyashio intrusion changes as does the outflow pattern of the Tsugaru Warm Current^29,32,57,58^. Among the three northern regions in our study, the southern limit of the first Oyashio intrusion—that is, the first branch of the Oyashio southward migration—is closest to Rikuchu and farthest from eastern Hokkaido^32^. The three southern regions are strongly influenced by the Kuroshio. The Kuroshio also shows marked path fluctuations; the variability of the distance between the three southern regions and the main Kuroshio axis is smallest at Osumi, followed by Boso, with the greatest variability at Nanki^27,31^. In March 2011, the Rikuchu census area subsided 50 cm and experienced a tsunami, with local wave heights of 8–15 m, due to the Great East Japan Earthquake^45^.

Within each region, we chose five shores at intervals of 2.7–17 km (8.2 ± 4.3 km)^52^ along the coastline (Fig. 4b). Within each shore, we established five 5000-cm^2^ permanent census plots (the total number of plots was 150) on steep rock walls in semi-exposed locations at intervals of 3.1–378 m (37.3 ± 48.9 m)^52^. Each plot was 50 cm wide by 100 cm high, with the vertical midpoint corresponding to mean tidal level. The vertical extent of the permanent plots covered 72.4% of the tidal range (138.2 cm between mean high water and mean low water of spring tides)^45^. The angles of the rock walls in the plots varied between 41° and 103° (71.6° ± 15.8°)^52^ from horizontal (0°). Although the slopes varied across sites, most census sites (all plots except three) had slopes of 50°–100°, neither moderate nor overhanging. Detailed study-site descriptions and biogeographic features of the area are presented elsewhere^40,41^. In each census plot, the presence or absence of all sessile species within a quadrat was recorded by eye, whereas coverage of sessile organisms was determined by counting their occurrence at 200 grid points at intervals of 5 cm both vertically and horizontally from 2003 to 2018. From 2007 on, we were unable to survey 9 out of 150 plots, for example those buried by sediments.

### Data analysis

#### Community trajectory analysis

We quantified rates and patterns of community dynamics following methods described elsewhere^5^. From presence/absence data for sessile organisms in individual census plots, we calculated the Sørensen dissimilarity index as a time series for successive years relative to the initial year. For both presence/absence and coverage data, we excluded rare taxa that accounted for < 2% of records in each region during the survey period. The frequency of occurrence of each taxon of sessile organism in each region is in Supplementary Table S2 online. We next fitted the dissimilarity estimates from each census plot to mathematical functions defining each trajectory (i.e., we determined the model coefficients of each trajectory).

Finally, we judged the models’ goodness-of-fit with the concordance criterion (CC), which measures the level of agreement between observed and predicted values for linear and nonlinear models. CC ≤ 0 indicates lack of fit, and CC = 1 indicates perfect fit, where

5 $${\text{CC}}_{i} = 1 - \frac{{\mathop \sum \nolimits_{j = 1}^{{n_{i} }} \left( {y_{ij} - \hat{y}_{ij} } \right)^{2} }}{{\mathop \sum \nolimits_{j = 1}^{{n_{i} }} \left( {y_{ij} - \overline{y}} \right)^{2} + \mathop \sum \nolimits_{j = 1}^{{n_{i} }} \left( {\hat{y}_{ij} - \overline{{\hat{y}}} } \right)^{2} + n_{i} \left( {\overline{y} - \overline{{\hat{y}}} } \right)^{2} }}.$$

Here $\overline{y}$ and $\overline{{\hat{y}}}$ are the means of observed (*y*_*ij*_) and predicted ($\overline{{\hat{y}}}_{ij}$) values for sample *i*, respectively, and *n*_*i*_ is the sample-specific number of observations in the time series.

We judged parsimony by using the Akaike information criterion (AIC); competing models with ΔAIC ≤ 2 were included as trajectories for a community. We first checked whether the fitted model yielded meaningful coefficient estimates. Here, we verified if the coefficients were of the correct sign and distinguishable from zero at *P* = 0.10 (e.g., whether α > 0 for the fitted linear model). Second, we checked if the model with the highest CC was also supported by the AIC. If not, we checked the model with the second-highest CC and its corresponding AIC. If no winning model emerged at this third step, we then considered the plot “unresolved”. This three-step screening to identify the best-fit model ensures that (1) a model is built with meaningful coefficients, (2) the coefficients well-describe the data, and (3) all coefficients are necessary. From the coefficients, CC, and AIC of each model, we classified the trajectory for each plot as stable, reversible, abrupt, or linear. Detailed descriptions of this method are available elsewhere^5^.

#### Temporal community invariability

We estimated the temporal invariability of species richness, species composition, and community abundance for each plot. Temporal invariability of species richness was calculated as the inverse of the coefficient of variation (CV) of species richness from presence/absence data. Temporal invariability of species composition was the mean of the Jaccard similarity in sessile community composition (calculated from species presence/absence data) between consecutive years^10,59^. Finally, temporal invariability of community abundance was calculated from coverage data as the inverse of the CV of total sessile community coverage^9,50^.

### Statistical analysis

To detect regional differences in the proportional distribution of trajectories, we performed Fisher’s exact test by using Monte Carlo simulations with 10,000 iterations. The following analyses were applied to local communities with linear or stable trajectories because our trajectory analyses showed that most correctly categorized trajectories were linear or stable (Fig. 2). To evaluate how community stability varied among regions within each trajectory, we performed one-way analysis of variance (ANOVA), in which each coefficient estimate of the best-fit model was treated as a response variable, and region was a fixed factor. To evaluate how temporal invariability of species richness, species composition, and community abundance varied among regions and trajectories, we performed two-way ANOVA, in which the response variables were (1) the inverse of the CV of species richness, (2) the mean of the Jaccard similarity between consecutive years, and (3) the inverse of the CV of total sessile community cover, as indicators of temporal invariability, and region and trajectory (linear or stable) were fixed factors. For significant ANOVAs (*P* < 0.05), we performed post hoc Bonferroni pairwise comparisons to evaluate differences between significant groups.

To examine how differences in current systems and the strength of current fluctuations influenced trajectories and temporal invariability of species richness, species composition, and community abundance, we performed multiple regression analyses, in which each trajectory coefficient and the three measures of temporal invariability were treated as response variables, and current systems and current fluctuations were explanatory variables. Here, current system was defined as a dummy variable (Kuroshio = 0, Oyashio = 1), and current fluctuations in each current system were assigned categorical values in descending order of fluctuation in the flow patterns of the ocean current (eastern Hokkaido = 1, southern Hokkaido = 2, Rikuchu = 3, Boso = 2, Nanki = 3, and Osumi = 1). All ANOVAs and multiple regressions were performed after checking the homogeneity of variance of the data (Levene’s test) and applying the appropriate transformation. To evaluate the interdependence among the three measures of temporal community invariability, we calculated Spearman's rank correlation coefficients. All statistical analyses were executed with R version 3.5.2^60^.

## Supplementary Information

Supplementary Table S1.

Supplementary Table S2.

Supplementary Figure S1.

Supplementary Information 1.

Supplementary Information 2.

Supplementary Information 3.

Supplementary Information 4.

Supplementary Information 5.

Supplementary Information 6.

Supplementary Information 7.

Supplementary Information 8.

Supplementary Legends.
