# Supplementary material for: Quantifying the dynamics of rocky intertidal sessile communities along the Pacific coast of Japan: implications for ecological resilience
Source: Sci Rep. 2021 Aug 9;11:16073. doi: 10.1038/s41598-021-95348-1 (PMC8352913; doi:10.1038/s41598-021-95348-1)
Supplement: Supplementary file 1 — Supplementary Table S1. [file 41598_2021_95348_MOESM1_ESM.pdf]

Table S1. Results of ANOVAs for the effect of region on model coefficients of each trajectory type (linear [a], stable [b and c]) of rocky intertidal sessile communities in six regions along the Pacific coast of Japan.

| Source of variation | df | MS | *F* | *P* |
|---|---|---|---|---|
| **(a) α, coefficient of the linear trajectory** | | | | |
| Region | 4 | 0.00006155 | 3.163 | 0.032 |
| Residuals | 24 | 0.00001946 | | |
| Transformation: none | | | | |
| Levene's test: $F$ = 1.9349, $P$ > 0.05 | | | | |
| | | | | |
| **(b) $\varphi_1$, coefficient of the stable trajectory** | | | | |
| Region | 5 | 0.05187 | 11.490 | <0.001 |
| Residuals | 67 | 0.00451 | | |
| Transformation: none | | | | |
| Levene's test: $F$ = 0.5406, $P$ > 0.05 | | | | |
| | | | | |
| **(c) $\varphi_2$, coefficient of the stable trajectory** | | | | |
| Region | 5 | 0.9775 | 5.364 | <0.001 |
| Residuals | 67 | 0.1822 | | |
| Transformation: none | | | | |
| Levene's test: $F$ = 0.7071, $P$ > 0.05 | | | | |
