# Supplementary material for: Quantifying the dynamics of rocky intertidal sessile communities along the Pacific coast of Japan: implications for ecological resilience
Source: Sci Rep. 2021 Aug 9;11:16073. doi: 10.1038/s41598-021-95348-1 (PMC8352913; doi:10.1038/s41598-021-95348-1)
Supplement: Supplementary file 12 — Supplementary Legends. [file 41598_2021_95348_MOESM12_ESM.docx]

**Supplementary information**

Table S1. Results of ANOVAs for the effect of region on model coefficients of each trajectory type (linear [a], stable [b and c]) of rocky intertidal sessile communities in six regions along the Pacific coast of Japan.

Table S2. Frequency of occurrence of each sessile organism in each region.

Fig. S1. Spearman rank correlation coefficients (*r*) for the relationships between three measures of community temporal invariability.

Supplementary Material 1. Data for Supplementary Table S1 and Table 2.

Supplementary Material 2. Data for Fig. 2 (including 6 files).

Supplementary Material 3. Data for Fig. 3 and Supplementary Fig. S1.
